# Associations within school-based same-sex friendship networks of children’s physical activity and sedentary behaviours: a cross-sectional social network analysis

**DOI:** 10.1186/s12966-018-0653-9

**Published:** 2018-02-21

**Authors:** Ruth E. Salway, Simon J. Sebire, Emma Solomon-Moore, Janice L. Thompson, Russell Jago

**Affiliations:** 1 Centre for Exercise, Nutrition & Health Sciences, School for Policy Studies, University of Bristol, 8 Priory Road, Bristol, BS8 1TZ UK; 2 Department for Health, University of Bath, Claverton Down, Bath, BA2 7AY UK; 3 School of Sport, Exercise and Rehabilitation Sciences, University of Birmingham, Birmingham, B15 2TT UK

**Keywords:** Social network, Peers, Physical activity, Sedentary time

## Abstract

**Background:**

Physical activity in children is associated with better physical and mental health but many children do not meet physical activity guidelines. Friendship groups are potentially an important influence on children’s physical activity and sedentary time. This paper examines the association between children of physical activity and sedentary time in school-based same-sex friendship networks, for both moderate-to-vigorous intensity physical activity (MVPA) and sedentary time. Moreover, considering the methodological challenges of conducting and interpreting these analyses, we provide examples of how to analyse these data and interpret results to encourage further work in the area.

**Methods:**

Accelerometer data for 1223 children, aged 8-9 years, were collected in 2015-2016 and analysed in 2017. Mean accelerometer minutes of MVPA and sedentary time were calculated. Children named up to four school friends and same-sex school-based friendship networks were constructed. Network models, which include correlation between friends, were fitted by sex.

**Results:**

Both MVPA and sedentary time were found to be associated via the friendship networks, for both boys and girls. The network autocorrelation was 0.21 (95% CI: 0.15 to 0.26) for boys’ MVPA, and 0.14 (95% CI: 0.07 to 0.21) for sedentary time. Network autocorrelation between girls was weaker, with 0.13 (95% CI: 0.06 to 0.19) for MVPA and 0.11 (95% CI: 0.05 to 0.17) for sedentary time.

**Conclusions:**

Physical activity and sedentary time of boys and girls are associated with the physical activity and sedentary time respectively of others within same-sex friendship networks, and these associations are comparable to other known factors. In this study, the correlation between friends was stronger for boys than girls, and stronger for MVPA than for sedentary time. These findings suggest that friendship networks play a part in understanding children’s physical activity and sedentary time and could play a valuable role in developing effective interventions.

**Electronic supplementary material:**

The online version of this article (10.1186/s12966-018-0653-9) contains supplementary material, which is available to authorized users.

## Background

Among children, physical activity is associated with lower levels of cardiometabolic risk factors and improved psychological well-being [1]. High levels of sedentary time have been associated with increased levels of cardiometabolic risk factors among children, but it is uncertain whether these effects are independent of physical activity [2–4]. It is recommended that all children and adolescents engage in at least 60 min of moderate-to-vigorous-intensity physical activity (MVPA) per day and limit their sedentary time [5]. However, a number of national surveys suggest that many children and young people do not meet physical activity guidelines, with girls less active than boys at all ages [6, 7]. For example, data from the nationally-representative Millennium cohort in the UK showed that among 7-8-year-old boys 63% met the physical activity hour per day recommendation and spent on average 6.4 h in sedentary time, while only 38% of girls achieved the recommendation and spent an average of 6.5 h in sedentary time [6]. Similarly, data from National Health and Nutrition Examination Surveys in the US estimates that for 6-11-year-olds, only 46% of boys and 22% of girls meet the recommendation [7]. There is a need to find ways to help children be more active and less sedentary.

Levels of physical activity and sedentary time amongst close friends may be an important influence on children’s physical activity [8–11] but this has been relatively under-explored. To date, a few studies have examined associations between physical activity in children and the physical activity of specific friends [12, 13] or the proportion of friends who are active [14, 15], but these do not take into account the more complex wider network of friends, or allow for dependence between them, which can result in biased parameter estimates. Social network analysis techniques have been used to model the full friendship network structure and include the dependence, and can be differentiated into those that focus on a child’s physical activity as the outcome measure (and use the friendship network to describe how one child’s activity relates to others’) [16, 17], and those that focus on modelling the formation of friendship ties (and include physical activity levels as an explanatory factor) [18–21]. These studies all show weak-to-moderate associations between children’s MVPA levels, and indicate possible differences between girls and boys, although they are inconsistent on whether associations are stronger for girls or boys. However, some of these studies rely on self-reported measures of activity [14, 15, 17–19], the majority are in adolescents rather than younger children [12, 14, 15, 17–19] and none of them have examined sedentary time. As a result, there is a paucity of studies that correctly model these complex relationships with objectively-measured activity data.

The aim of this paper is to examine the association between children of physical activity and sedentary time in same-sex school-based friendship networks both for MVPA and sedentary time. Previous evidence suggests differences between girls and boys in terms of their levels of physical activity and sedentary time, with girls generally less active and more sedentary, so we are interested in whether associations within friendship networks also differ by sex. Correctly modelling friendship networks that involve dependence between children is methodologically challenging, and so we additionally aim to provide examples of how to analyse these data and interpret results to encourage further work in the area. (Technical details are given in Additional file 1).

## Methods

Data are from the B-PROACT1V study, which aimed to examine the physical activity behaviours of primary school children, aged 5-11 years, and their parents (described in detail elsewhere [22–24]). The study received ethical approval from the School for Policy Studies Ethics Committee at the University of Bristol, UK, and written parental consent was received for all participants [25]. This analysis uses data collected between March 2015 and July 2016, from Phase 2, where all children in Year 4 of primary school (aged 8-9 years) from 47 schools in and around Bristol, were invited to participate. Of these, 59.7% were given parental consent, resulting in 1223 children with data.

### Child accelerometer measures

Children wore a waist-worn ActiGraph wGT3X-BT accelerometer for five days, including two weekend days. Accelerometer data were processed using Kinesoft (v3.3.75; Kinesoft, Saskatchewan, Canada) and analysis was restricted to those children who provided at least three days of valid data (91% provided at least one valid weekend day). A valid day was defined as at least 500 min of data, after excluding intervals of ≥60 min of zero counts allowing up to 2 min of interruptions. Data were recorded at 10 s intervals and characterised as sedentary, light or MVPA using Evenson population-specific cut points for children [26]. The average number of MVPA and sedentary minutes per day were derived for each child.

### Friendship networks

Children were asked to name up to four of their closest friends within their school and year group. These nominations were matched with other participants in the study to develop friendship networks. A total of 4612 friends were nominated, of whom 3117 (68%) were in the study (the median number of friends was 3). As the focus of this paper is to explore differences between girls and boys, we restrict analysis to same-sex friendship networks, and so 313 (10%) friendships ties between children of the opposite sex and 87 (7%) children who had no same-sex friends taking part in the study were removed from the analysis.

### Other measurements

We considered body mass index (BMI) and area deprivation to be potential confounders as these might influence activity levels in individual children and across children in networks. Child height and weight were measured, and child BMI was calculated and converted to an age- and sex-specific standard deviation score [27, 28]. Indices of Multiple Deprivation (IMD) scores, based on the English Indices of Deprivation (http://data.gov.uk/dataset/index-of-multiple-deprivation), were assigned to each child based on their reported home postcode. Higher IMD scores indicate a greater level of deprivation. Children completed a short questionnaire, in which they were asked about the frequency (coded from 0 = ‘Never’ to 3 = ‘5 days per week’) with which they engaged in different forms of activity outside school hours: sport or exercise club at school, sport or exercise club elsewhere, playing outdoors in their neighbourhood, and playing outdoors at home. These were combined to form a covariate of activity participation score from 0 to 12, with a higher value indicating a higher frequency of participation in activities outside school [29]. This activity participation variable provides information on the type of activity rather than intensity, and has been shown to be an important predictor of activity with patterns that differ between girls and boys [29].

### Statistical analysis

#### Missing data

Missing data were imputed for accelerometer measurements and all covariates. Missing data varied from 0.5% for BMI z-score to 18% for sedentary time, with a total of 433 (78%) of the 556 boys and 540 (81%) of the 667 girls having complete data. Any child with fewer than three valid days of accelerometer data had their accelerometer measures imputed. Multiple imputation methods were used, to create 20 imputed datasets each for boys and girls separately, using 20 cycles of regression switching and combined regression coefficients across datasets using Rubin’s rules [30]. All exposures, outcomes and potential confounders, including the child’s school, were included and the activity participation variable was imputed passively. Subsequent analyses were run by sex.

#### Friendship networks

**Fig. 1:**
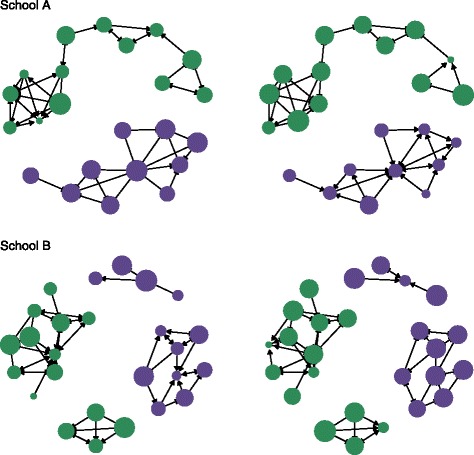
Network plots of average MVPA (left) and sedentary time (right) for two typical schools (top and bottom). *Legend*: Nodes represent individual children, and are sized by average minutes of MVPA (left) and average minutes of sedentary time (right) and coloured purple for boys and green for girls. The same child is in the same position in both plots

Friendship networks for boys and girls were plotted for each school (Fig. 1), with each node representing a child and links between nodes representing friendship ties. Nodes were scaled by mean MVPA and mean sedentary time to assess the extent to which similar nodes clustered together. Moran scatter plots [31] were plotted for mean MVPA and mean sedentary time to compare a child’s MVPA or sedentary time with the average MVPA or sedentary time of their friends. This graph provides a visual representation of how similar children are to their immediate friends and gives an indication of the direction and strength of autocorrelation within the full friendship networks.

#### Model comparison

Standard linear OLS regression models were run by sex for mean MVPA and mean sedentary time with BMI z-score, IMD deprivation score and the activity participation score included as covariates. This model was interpreted as the baseline model for comparison purposes. Moran’s I statistics were calculated for the residuals to assess the extent of any autocorrelation between the MVPA or sedentary time of children within a friendship network after taking into account the covariates.

To estimate the extent to which children’s MVPA, and sedentary time, were correlated via the friendship network structure, network autocorrelation models, also known as network effect or spatial lag models, were fitted for mean MVPA and mean sedentary time (centred around the mean) (see Additional file 1 for further details). The network structure for boys and girls separately was described using a contiguity adjacency matrix, standardised so that all non-zero rows sum to 1 [32]. Models were stratified by sex, so the network autocorrelation parameter represents dependence between children of the same sex. Because of the correlation introduced via the friendship network, interpretation of the coefficients in the network model is more complex and further details are given in Additional file 1.

The network models were descriptively compared to the baseline models. The Akaike Information Criterion (AIC) [33] was calculated to assess model fit, with a lower value indicating a better-fitting model. All analyses were performed in 2017 using R version 3.3.3 [34], using multiple imputation and adjusting the standard errors to account for clustering of children within schools.

## Results

There were 1136 participants aged 8-9 years with same-sex friends in the study, of whom 506 (45%) were boys and 630 (55%) girls. (Additional file 2: Table S1 and S2) show the characteristics of the children split by sex for the imputed and observed datasets. All characteristics had similar distributions for the imputed and observed data. Boys engaged in more daily minutes of MVPA than girls (69.7 mins vs. 55.6 mins, and spent less time being sedentary (424.8 mins vs. 439.0 mins). Boys’ activity participation score was slightly higher than girls’ (6.2 vs. 5.6), but there was no difference in BMI z-score (0.26 vs 0.39) or IMD score (15.0 vs. 16.1).

Figure 1 shows network plots of mean MVPA and sedentary time for two typical schools. Nodes tend to be linked to similar-sized nodes, indicating that children tend to be friends with children of similar MVPA or sedentary time. Similar patterns were evident for the majority of schools.

Moran scatter plots for mean MVPA and sedentary time (Additional file 2: Figure S1) show positive associations between a child’s MVPA and sedentary minutes and those of their immediate friends. Boys’ MVPA showed a stronger association with friends’ levels (of the same sex) than did girls’ MVPA with their friends’. Sedentary time associations were weaker than those for MVPA and appeared more similar in boys and girls. Moran’s I statistics were calculated for the residuals from the baseline model (Additional file 2: Table S3). A value close to 1.0 indicates a high level of correlation between children within a friendship network. Boys’ MVPA showed the highest correlation (I = 0.204, *p* < 0.0005), followed by boys’ sedentary time (I = 0.127, *p* = 0.006). Evidence for correlation within girls’ MVPA and sedentary time was weaker (I = 0.096, *p* = 0.009 for MVPA, I = 0.088, *p* = 0.017 for sedentary time). These results indicate that MVPA and sedentary time are correlated within friendship networks, and that modelling this correlation is therefore appropriate in all cases.

The baseline and network models for mean MVPA are summarised in Tables 1 and 2 for boys and girls, respectively. For the network models, the network autocorrelation measures the strength of correlation between the MVPA of children within a friendship network, adjusting for covariates. The network autocorrelation was 0.21 (95% CI: 0.15 to 0.26) for boys and 0.13 (95% CI: 0.06 to 0.19) for girls. This indicated a positive association between the mean MVPA of child within their friendship networks, with a stronger association for boys than for girls. For both sexes, the AIC for the network effects model was lower than for the baseline model indicating that inclusion of the network effect term improved model fit.

**Table 1:** Comparison of baseline OLS regression with network model for average MVPA minutes (boys)

			Change in mean MVPA (mins) for a one unit increase in covariate					
	coefficient	95% CI	Direct	95% CI^a^	Indirect ‘Spillover’	95% CI^a^	Total	95% CI^a^
Baseline OLS regression model (*n* = 556)								
Constant	−14.57	(−23.10, −6.04)						
IMD	−0.04	(−0.30, 0.23)						
BMI z-score	−2.51	(−4.79, −0.24)	− 2.51	(− 4.79, − 0.24)	0		−2.51	(− 4.79, − 0.24)
Activity participation score	2.55	(1.62, 3.48)	2.55	(1.62, 3.48)	0		2.55	(1.62, 3.48)
AIC = 4657.6								
Network model (*n* = 556)								
Constant	−14.76	(−22.56, −6.96)						
IMD	−0.004	(−0.24, 0.24)						
BMI z-score	−2.64	(−4.77, −0.50)	− 2.68	(− 4.84, − 0.51)	−0.63	(−1.21, − 0.06)	−3.31	(−6.01, − 0.61)
Activity participation score	2.45	(1.57, 3.33)	2.48	(1.59, 3.37)	0.59	(0.29, 0.89)	3.07	(1.94, 4.21)
Network dependence	0.21	(0.15, 0.26)						
AIC = 4637.0								

^a^Impact CI based on 2.5 and 97.5 percentiles of 200 simulations

**Table 2:** Comparison of baseline OLS regression with network model for average MVPA minutes (girls)

			Change in mean MVPA (mins) for a one unit increase in covariate					
	coefficient	95% CI	Direct	95% CI^a^	Indirect ‘Spillover’	95% CI^a^	Total	95% CI^a^
Baseline OLS regression model (*n* = 667)								
Constant	−8.57	(−13.24, −3.89)						
IMD	0.005	(−0.15, 0.16)						
BMI z-score	− 0.81	(−2.19, 0.58)						
Activity participation score	1.57	(0.80, 2.33)	1.57	(0.80, 2.33)	0		1.57	(0.80, 2.33)
AIC = 5426.5								
Network model (*n* = 667)								
Constant	−8.57	(−13.06, −4.07)						
IMD	0.01	(−0.13, 0.16)						
BMI z-score	−0.84	(−2.19, 0.50)						
Activity participation score	1.54	(0.79, 2.28)	1.54	(0.79, 2.30)	0.21	(0.05, 0.37)	1.76	(0.89, 2.63)
Network dependence	0.13	(0.06, 0.19)						
AIC = 5420.3								

^a^Impact CI based on 2.5 and 97.5 percentiles of 200 simulations

For boys, both the baseline and network models indicated that mean MVPA was positively associated with activity participation score, and negatively associated with BMI z-score. For girls, only activity score was predictive of mean MVPA in both models. Interpretation of the coefficients in the network model is more complex than for a standard regression model, because of the dependence between outcomes. Briefly, any change in the independent variable is related to a direct impact on the child’s MVPA (or sedentary time) plus an indirect impact on the MVPA (or sedentary time) of other children in the network; a fuller explanation, with an example, is given in Additional file 2, and direct, indirect and total impacts are reported in Tables 1, 2, 3 and 4.

**Table 3:** Comparison of baseline OLS regression with network model for average sedentary time minutes (boys)

			Change in mean sedentary time (mins) for a one unit increase in covariate					
	coefficient	95% CI	Direct	95% CI^a^	Indirect ‘Spillover’	95% CI^a^	Total	95% CI^a^
Baseline OLS regression model (*n* = 556)								
Constant	20.98	(0.72, 41.23)						
IMD	0.15	(−0.48, 0.78)						
BMI z-score	2.02	(−3.32, 7.36)						
Activity participation score	−3.84	(−6.41, −1.27)	−3.84	(−6.41, −1.27)	0		−3.84	(−6.41, −1.27)
AIC = 5609.6								
Network model (*n* = 556)								
Constant	21.15	(2.57, 39.72)						
IMD	0.16	(−0.40, 0.72)						
BMI z-score	1.76	(−3.37, 6.89)						
Activity participation score	−3.86	(−6.31, −1.40)	−3.88	(−5.14, −2.62)	−0.58	(− 0.93, − 0.23)	−4.46	(−5.89, − 3.04)
Network dependence	0.14	(0.07, 0.21)						
AIC = 5602.2								

^a^Impact CI based on 2.5 and 97.5 percentiles of 200 simulations

**Table 4:** Comparison of baseline OLS regression with network model for average sedentary time minutes (girls)

			Change in mean sedentary time (mins) for a one unit increase in covariate					
	coefficient	95% CI	Direct	95% CI^a^	Indirect ‘Spillover’	95% CI^a^	Total	95% CI^a^
Baseline OLS regression model (*n* = 667)								
Constant	12.11	(−4.95, 29.17)						
IMD	0.01	(−0.44, 0.46)						
BMI z-score	1.00	(−3.89, 5.89)						
Activity participation score	−2.25	(−4.74, −0.24)						
AIC = 6932.6								
Network model (*n* = 667)								
Constant	11.89	(−4.68, 28.45)						
IMD	−0.01	(−0.42, 0.41)						
BMI z-score	1.01	(−3.78, 5.81)						
Activity participation score	−2.16	(−4.62, 0.31)						
Network dependence	0.11	(0.05, 0.17)						
AIC = 6928.4								

^a^Impact CI based on 2.5 and 97.5 percentiles of 200 simulations

The baseline and network models for mean sedentary time are summarised by sex in Tables 3 and 4. The network autocorrelation parameter was 0.14 (95% CI: 0.07 to 0.21) for boys and 0.11 (95% CI: 0.05 to 0.17) for girls, indicating a weak positive correlation between mean sedentary times across friendship networks, for both boys and girls. This association was weaker than that seen for MVPA. Again, comparison of AIC for the network effect and baseline models suggested that inclusion of the network autocorrelation term improved model fit slightly. Activity participation score was predictive of mean sedentary time for boys, with a negative association between activity and sedentary time. For girls, activity participation score had no impact on sedentary time.

All patterns were broadly comparable when re-run using the complete data only (Additional file 2: Table S4).

## Discussion

Physical activity and sedentary time of boys and girls were associated with the physical activity and sedentary time of others in the same-sex friendship network. Network autocorrelations were small, indicating that the majority of dependence was between children and their immediate friends rather than more distant friendships. The strongest associations were between boys’ MVPA, followed by boys’ sedentary time, with weaker associations for girls’ MVPA and sedentary time. No clear guidelines for interpreting autocorrelations exist, although correlations of this size would not generally be considered strong. However, relatively few factors have been consistently identified as correlates of physical activity [35] or sedentary time [36], and the autocorrelations found here represent moderate associations when compared to correlations with other known factors. For example, in our dataset, the correlation between BMI z-score and MVPA was − 0.08 and between IMD score and MVPA was − 0.06, compared to autocorrelations for MVPA of 0.21 (boys) and 0.13 (girls).

We saw stronger associations within friendship networks among boys than girls, and for physical activity than for sedentary time. Many studies show that boys tend to be more active than girls [6], and these results show that they are also more likely to be active with friends. Sedentary time is typically associated with different factors to MVPA [35, 36], with differences by gender less evident, and these results show similar patterns for friendship networks; associations are similar for girls and boys, and sedentary time is less strongly associated with friendship networks than MVPA. This is perhaps as being active tends to involve activities that are done with or alongside others, whereas sedentary behaviours are often individual or solitary.

Our results show different associations for boys and girls, and for MVPA and sedentary time, which is typical of studies generally in this area. Although research on physical activity and sedentary time within friendship groups in this age group is limited, these results are broadly consistent with previous research which found a similar-sized network autocorrelation for MVPA for boys and girls together [16], and an association between MVPA and best friend’s MVPA for boys, but not for girls [13]. Other studies [12, 14, 17] have found evidence of associations with friends’ activity in older adolescents, although the measures and methods used are not directly comparable to those used here. Our analysis models the complex dependence between children via the friendship network, which allows us to estimate the extent of that dependence, and extends previous analysis to include sedentary time. The results support the conclusion that there may be differences in physical activity between boys and girls that are at least partially a function of their network, and adds evidence that sedentary behaviour is distinct from physical activity in that it shows a weaker association with friendship networks than MVPA.

Results suggest that friendship networks play a part in understanding factors that are associated with children’s physical activity and sedentary time that is comparable to other known factors, and underline the benefits of further use of social network analysis techniques in studies of children’s physical activity to correctly model dependence between friends. Developing interventions that increase physical activity or reduce sedentary time in children is important for tackling long-term health problems, and understanding the relationships of physical activity and sedentary time within friendship networks could play a valuable role. This role may be indirect as any intervention that increases a child’s physical activity or reduces sedentary time may also have an effect on others in the network, or it may be direct by developing interventions designed to explicitly target friendship groups. In the latter case, our results suggest that interventions aimed at increasing physical activity among boys might particularly benefit from utilising friendship networks.

### Strengths and limitations

The analysis presented here offers several advantages in understanding associations between friends. The network model accounts for correlation between both physical activity and sedentary time across the friendship network structure, which exhibits improved model fit when network correlation is present and allows us to estimate the strength of that correlation. It offers flexibility in the form of the network structure via the weight matrix, and distinguishes between one-way and reciprocal friendship ties. Omitting the network dependence not only excludes important information about the influences on children’s physical activity, but can also affect conclusions about other covariates through biased parameter estimates and the width of confidence intervals. In addition, the analysis uses objectively-measured accelerometer data for all children rather than self-reported activity measures or reported friendship activity, and bias due to missing data is reduced by using multiple imputation.

This analysis uses cross-sectional data, so although we have shown an association between a child’s physical activity and sedentary time and that of their friends, we cannot distinguish whether this is because children tend to form friendships with those of a similar activity level, or whether children tend to adjust their levels of physical activity to match those of their friends. In order to explore differences between boys and girls we restricted the networks to same-sex friendships, and so our analysis may miss any differences in correlation between friends of opposite sex, although these form only a minority of friendships.

Social network techniques are susceptible to bias due to missing data about the structure of the network [37]. Specifically, our analysis excludes friendships outside of school, and any friends nominated who were not participating in the study. Children were only able to nominate a maximum of four friends and the majority used all four of their nominations, suggesting that we may be missing information about further friendships. However, the network model involves aggregate measures of activity over the friendship network, and as such is less sensitive to missing activity data within the network. There is evidence to suggest that the network autocorrelation parameter in these models is underestimated [38, 39], especially for large parameters and very dense networks, so our model may underestimate the strength of the association.

## Conclusions

Physical activity and sedentary time of children are associated with the physical activity and sedentary time of others within the same-sex friendship network, and these associations are comparable to other known factors. We saw stronger associations within friendship networks among boys than girls, and for physical activity than for sedentary time.

Understanding the relationships of physical activity and sedentary time within friendship networks could play a valuable role in developing effective interventions, especially those that target increasing physical activity among boys.

## Additional files

Additional file 1: Details and interpretation of network autocorrelation model. (DOCX 43 kb)

Additional file 2: Additional Tables and Figures. **Table S1.** Characteristics of children included in the analysis, including imputed values. **Table S2.** Characteristics of children included in the analysis, observed data only. **Table S3.** Moran’s I statistic for network autocorrelation in the residuals from the baseline OLS regression models. **Figure S1.** Moran plots for MVPA and sedentary time. (DOCX 48 kb)

## Abbreviations

AIC: Akaike Information Criterion
BMI: Body Mass Index
IMD: Index of Multiple Deprivation
IQR: Inter-quartile range
MVPA: Moderate to Vigorous intensity Physical Activity
OLS: Ordinary least squares
SD: Standard deviation
UK: United Kingdom
