# Supplementary material for: Associations within school-based same-sex friendship networks of children’s physical activity and sedentary behaviours: a cross-sectional social network analysis
Source: Int J Behav Nutr Phys Act. 2018 Feb 21;15:18. doi: 10.1186/s12966-018-0653-9 (PMC5822654; doi:10.1186/s12966-018-0653-9)
Supplement: Supplementary file 1 — Details and interpretation of network autocorrelation model. (DOCX 43 kb) [file 12966_2018_653_MOESM1_ESM.docx]

**Additional File 1: Details and interpretation of network autocorrelation model**

## Network Autocorrelation Model

This paper uses a network autocorrelation model (sometimes known as a network effect model) to describe the dependence between the physical activity of children within a friendship network. These models are based on models in the spatial statistics literature, but instead of relationships acting over physical space, they act over the network structure. As this model is little used in the area of children’s physical activity, this Appendix gives some technical details of the model, how to fit it and how to interpret it.

A standard linear regression model, with independent outcomes, is given by:

|  | $\begin{matrix} y=X\beta+\varepsilon, & \varepsilon∽N\left( 0, \sigma^{2}I \right) \end{matrix}$ |  | (1) |
| --- | --- | --- | --- |

where $y$ is the outcome variable (physical activity in our case), $X$ is the set of independent variables, including an intercept term, to be included in the model, $\beta$ is the vector of parameter estimates and $\varepsilon$ are error variables which are assumed to be independent identically-distributed Normal random variables. In this model, the responses $y$ are independent of each other, and the friendship network plays no role.

### Network autocorrelation

There are two main ways of incorporating network autocorrelation, that is, dependence caused by the physical activity of children close together in the network being more strongly correlated, into this model.

The first is to extend the standard regression model (1) by adding a term that captures the contribution from other friends in the network:

|  | $\begin{matrix} y=\rho Wy+X\beta+\varepsilon, & \varepsilon∽N(0, \sigma^{2}I) \end{matrix}$ | (2) |
| --- | --- | --- |

This is known as the *network effect model* in the social network literature, or the *spatial lag model* in spatial statistics/statistical geography. The network term is $\rho Wy$, which is a combination of $\rho$, a parameter which captures the strength of the network autocorrelation, $W$ a weight matrix that describes the network structure and $y$, the physical activity of the friends. The network autocorrelation parameter $\rho$ is sometimes described as the network influence (although this term suggests a causal relationship that is not appropriate in an observational setting) and can be thought of as the *average level of dependence over the network structure*. Positive values of $\rho$ indicate positive correlation between individuals in the network, with larger values of $\rho$ indicating stronger network correlation. Negative $\rho$ is rare in practice. Under certain conditions (row standardisation – see below) the network autocorrelation parameter is bounded by 1, and can be interpreted in a similar way to a correlation coefficient.

Note that the physical activity, $y$, appears both as a response variable and as a predictor in model (2) because the friends are themselves respondents in the study. Model (2) can be rewritten as

|  | $\begin{matrix} y={(I-\rho W)}^{-1}X\beta+ \delta& \end{matrix}$ |  |
| --- | --- | --- |
|  | $\begin{matrix} \delta={(I-\rho W)}^{-1}\varepsilon, & \varepsilon∽N(0, \sigma^{2}I) \end{matrix}$ |  |

where the error term $\delta$ is no longer independent between children. Thus, model (2) introduces dependence in the response variable $y$ via the network and so cannot be estimated in the standard linear regression framework. This model is common in spatial statistics/statistical geography, where it is known as the *spatial lag model*, and appropriate software tools are typically found in spatial statistics packages (for example, sppack in Stata, or spdep in R).

One way to interpret the network effect model is that it describes how an index child’s physical activity depends on the physical activity of friends. However, this can be confusing as it suggests that the network parameter term can be interpreted (incorrectly) as the effect of physical activity of immediate friends on a child’s physical activity in the same way that one might talk about the effect of BMI on a child’s physical activity. However, a child’s physical activity cannot simultaneously be both an outcome and an exposure in the same model; this interpretation hides the fact that all the children in the study have correlated physical activity outcomes.

An alternative way to introduce the correlation in the network is to capture it directly in the error term:

|  | $\begin{matrix} y=X\beta+ \varepsilon& \end{matrix}$ |  |
| --- | --- | --- |
|  | $\begin{matrix} \varepsilon=\lambda W\varepsilon& \end{matrix}$ | (3) |

where the dependence is modelled directly through the error term. As before, $W$ is the weight matrix that describes the network structure, and $\lambda$ a parameter that captures the strength of the dependence in the error terms. This model can be thought of as modelling a child’s tendency to deviate from the norm in the presence of other children who are also deviating from the norm. This is known as the *network disturbances model*, and is related to the *spatial error model* in spatial statistics. Such a model might be more appropriate when dealing with more abstract concepts such as motivation or perception about physical activity.^1^ In the current context, however, we prefer the network effect model, as there is a clear physical interpretation of the network term in the context of shared physical activity via children playing or being active together. In situations where the choice is less clear, model-based diagnostics may be used to compare the two.^2, p155^

### Choice of weight matrix

Network autocorrelation models depend crucially on the choice of $W$, which is specified by the researcher, and represents the network structure. There are many ways in which this can be defined. The simplest weight matrix is the contiguity adjacency matrix:

$$W_{ij}=\left\{ \begin{matrix} 1 & \text{if child} i \text{nominates} j \text{as a friend} \\ 0 & \text{otherwise} \end{matrix} \right.$$

This assumes that the only direct links are between a child and the friends they have nominated, and the term $Wy$ is then the sum of physical activity outcomes for all the nominated friends. This matrix need not by symmetric; if child $i$ nominates $j$ as a friend, it does not necessarily follow that child $j$ nominates $i$. Although this weight matrix does not directly capture more distant relationships (such as friends of friends) there is still indirect dependence between them, that decreases with the number of friends separating them. It is common to standardise the weight matrix so that rows sum to one; this has the advantage that the network dependence parameter $\rho$is then bounded by 1. In this situation it also makes intuitive sense, as children may nominate different numbers of friends, and so the network term becomes an average physical activity over all the nominated friends.

Much of the literature that discusses possible choices of $W$ is in the spatial geography literature, where the weights are interpreted in terms of inverse distance between two areas, with closer areas having greater weight. This makes sense in a spatial context, as it gives the weight matrix a physical interpretation, but it is less clear how to define a concept of ‘social distance’, and not always easy to interpret the meaning of a weight matrix which is derived in such a way. Instead, it is preferable to think of the weight matrix as representing social influence directly, rather than relying on an indirect concept of social distance.

# **Interpretation of network model**

### Network autocorrelation parameter

The network autocorrelation parameter $\rho$ captures the average level of dependence between outcomes (here, children’s MVPA or sedentary time) over the friendship network structure. It is a measure of the degree to which the outcomes tend to be clustered together in the friendship network, and can be interpreted as the extent to which outcomes are correlated with each other. It is not correct to interpret the network autocorrelation parameter as the effect of a unit increase in average friendship physical activity, as the friends themselves appear as outcomes, so it is not possible to increase friends’ physical activity without manipulating the dependent variable.

A value of zero indicates no autocorrelation in the network, that is, that the outcomes are independent. Although in general $\rho$ may take values greater than 1, in this paper we use a row-standardised weight matrix and so $\rho<1$, with values closer to one indicating stronger autocorrelation.

### Coefficients of independent variables

The interpretation of coefficients for the independent variables from the network effect model is not straightforward, as the dependence structure of the network means that a change in one individual affects all the others. These changes will differ depending on where the individual is in the network, and who they are linked to. Instead, we look at the average change on children across the network (these are called impacts^2, p37^ in the terminology of LeSage and Pace).

To illustrate, consider the example of activity participation score from the boys’ MVPA model. In the baseline OLS model (that is, the standard regression model with no network structure), the activity participation score has a coefficient of 2.55. If we increase a boy’s activity participation by one unit (this corresponds roughly to one additional active session per week) then the model predicts that this boy’s MVPA will increase by a total of 2.55 minutes, but other children remain unaffected as they are all independent (Figure S3, left hand diagram).

In the network model, activity participation score has a coefficient of 2.45. If we increase a boy’s activity by one unit, then as well as the direct impact on the child in question, there will also be an indirect (‘spillover’) impact on other boys in the network, as a change in one boy’s MVPA will result in a change in others’ (Figure S3, right hand diagram). Thus, the changes propagate through the network, including feedback to the original child, with the impacts gradually decreasing depending on the strength of the network effect parameter. The direct impact on the child in question is a combination of the immediate effect of the increase in activity score plus the feedback effects which gives a direct impact of an increase of 2.48 minutes in MVPA. The indirect impacts on other children consist of knock-on effects, which amount to an indirect impact of 0.59 minutes on MVPA. The total impact is the sum of these, a total impact of 3.07 minutes of MVPA across all children in the network.

Tables 1-4 in the main paper give direct, indirect and total impacts for covariates in the network effect model; we calculate changes only for those covariates where the 95% confidence intervals of the coefficients do not include zero. For comparison, the direct impact of the baseline model is the model coefficient itself, and the indirect impact is zero.

Figure S3: Interpretation of direct and indirect impacts for baseline and network effect models


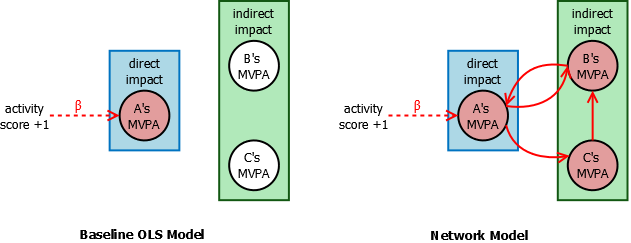


# **REFERENCES**

1. Leenders R. Modeling social influence through network autocorrelation: constructing the weight matrix. Soc Networks 2002;24:21-47.

2. LeSage JP, Pace RK. Introduction to Spatial Econometrics. CRC Press; 2009.
