# Supplementary material for: Associations within school-based same-sex friendship networks of children’s physical activity and sedentary behaviours: a cross-sectional social network analysis
Source: Int J Behav Nutr Phys Act. 2018 Feb 21;15:18. doi: 10.1186/s12966-018-0653-9 (PMC5822654; doi:10.1186/s12966-018-0653-9)
Supplement: Supplementary file 2 — Additional Tables and Figures. Table S1. Characteristics of children included in the analysis, including imputed values. Table S2. Characteristics of children included in the analysis, observed data only. Table S3. Moran’s I statistic for network autocorrelation in the residuals from the baseline OLS regression models. Figure S1. Moran plots for MVPA and sedentary time. (DOCX 48 kb) [file 12966_2018_653_MOESM2_ESM.docx]

**Additional File 2: Additional Tables and Figures**

Supplementary Table 1: Characteristics of children included in the analysis, including imputed values

Supplementary Table 2: Characteristics of children included in the analysis, observed data only

Supplementary Table 3: Moran's I statistic for network autocorrelation in the residuals from the baseline OLS regression models

Supplementary Table 4: network autocorrelation estimates (observed data only)

Supplementary Figure 1: Moran plots for MVPA and sedentary time

**Supplementary Table 1: Characteristics of children included in the analysis, including imputed values**

|  | | All (n=1136) | | Boys (n=506) | | Girls (n=630) | |
| --- | --- | --- | --- | --- | --- | --- | --- |
|  | | Mean/ median/ % | SD/  IQR | Mean/  median | SD/  IQR | Mean/ median | SD/  IQR |
| Sex | Boy | 45% |  |  |  |  |  |
|  | Girl | 55% |  |  |  |  |  |
| BMI z score | | 0.33 | 1.08 | 0.26 | 1.07 | 0.39 | 1.08 |
| IMD score | | 15.6 | 13.8 | 15.0 | 13.9 | 16.1 | 13.7 |
| Activity frequency score | | 5.9 | 2.3 | 6.2 | 2.4 | 5.6 | 2.2 |
| Average MVPA (mins/day) | | 61.9 | 22.6 | 69.7 | 24.9 | 55.6 | 18.2 |
| Average sedentary time (mins/day) | | 432.7 | 60.9 | 424.8 | 62.1 | 439.0 | 59.2 |
| Number of friends nominated ^a^ | | 3 | 1 | 2 | 1 | 3 | 1 |
| Number of nominations ^a^ | | 2 | 3 | 2 | 1 | 2 | 3 |
| ^a^  median/IQR  SD: standard deviation; IQR: inter-quartile range | | | | | | | |

**Supplementary Table 2: Characteristics of children included in the analysis, observed data only**

|  | |  | All | |  | Boys | |  | Girls | |
| --- | --- | --- | --- | --- | --- | --- | --- | --- | --- | --- |
|  | | n | Mean/ median/ % | SD/  IQR | n | Mean/  median | SD/  IQR | n | Mean/  median | SD/  IQR |
| Sex | Boy | 506 | 45% |  |  |  |  |  |  |  |
|  | Girl | 630 | 55% |  |  |  |  |  |  |  |
| BMI z score | | 1132 | 0.33 | 1.08 | 503 | 0.26 | 1.07 | 629 | 0.39 | 1.08 |
| IMD score | | 1118 | 15.6 | 13.8 | 500 | 15.0 | 13.8 | 618 | 16.2 | 13.7 |
| Activity frequency score | | 1117 | 5.9 | 2.3 | 495 | 6.2 | 2.4 | 622 | 5.6 | 2.2 |
| Average MVPA (mins/day) | | 962 | 61.7 | 22.0 | 418 | 69.4 | 24.3 | 544 | 55.8 | 18.0 |
| Average sedentary time (mins/day) | | 944 | 432.8 | 59.0 | 410 | 424.8 | 59.9 | 534 | 438.1 | 57.8 |
| Number of friends nominated ^a^ | | 1076 | 3 | 1 | 479 | 2 | 1 | 597 | 3 | 1 |
| Number of nominations ^a^ | | 1076 | 2 | 3 | 479 | 2 | 1 | 597 | 2 | 3 |
| ^a^  median/IQR  SD: standard deviation; IQR: inter-quartile range | | | | | | | | | | |

**Supplementary Table 3:** **Moran's I statistic for residual network autocorrelation in the baseline OLS regression models**

|  |  | Moran’s I | p-value^a^ |
| --- | --- | --- | --- |
| Average MVPA (mins) | Boys | 0.204 | **<0.005** |
|  | Girls | 0.096 | **0.009** |
| Average sedentary time (mins) | Boys | 0.127 | **0.006** |
|  | Girls | 0.088 | **0.017** |

Boldface indicates statistical significance (p<0.05)

^a^ Test for difference in Moran’s I statistic from expected mean of -0.003 under the null hypothesis of no network correlation

**Supplementary Table 4: network autocorrelation estimates (observed data only)**

|  | Boys | | Girls | |
| --- | --- | --- | --- | --- |
|  | Network autocorrelation | 95% CI | Network autocorrelation | 95% CI |
| MVPA | 0.26 | (0.23, 0.29) | 0.13 | (0.10, 0.17) |
| Sedentary time | 0.19 | (0.18, 0.21) | 0.13 | (0.11, 0.14) |

**Supplementary Figure 2: Moran plots for MVPA and sedentary time**


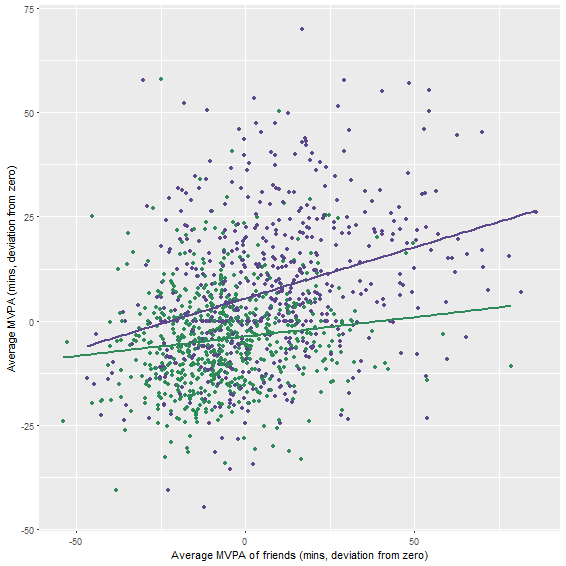

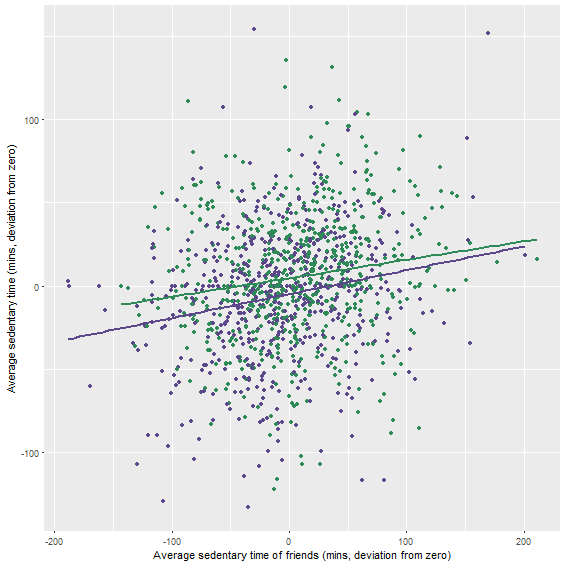


Note: Moran plots show the relationship between a child’s value and the average value of their immediate friends, centred to represent deviation from average. Boys = purple. Girls = green. Lines of best fit: boys=purple, girls = green
